# In silico identification of anti-cancer compounds and plants from traditional Chinese medicine database

**DOI:** 10.1038/srep25462

**Published:** 2016-05-05

**Authors:** Shao-Xing Dai, Wen-Xing Li, Fei-Fei Han, Yi-Cheng Guo, Jun-Juan Zheng, Jia-Qian Liu, Qian Wang, Yue-Dong Gao, Gong-Hua Li, Jing-Fei Huang

**Affiliations:** 1 State Key Laboratory of Genetic Resources and Evolution, Kunming Institute of Zoology, Chinese Academy of Sciences, Kunming 650223, Yunnan, China; 2 Kunming College of Life Science, University of Chinese Academy of Sciences, Beijing 100049, China; 3 Institute of Health Sciences, Anhui University, Hefei 230601, Anhui, China; 4 School of Life Sciences, University of Science and Technology of China, Hefei, Anhui 230027, China; 5 Kunming Biological Diversity Regional Center of Instruments, Kunming Institute of Zoology, Chinese Academy of Sciences, Kunming 650223, China; 6 KIZ-SU Joint Laboratory of Animal Models and Drug Development, College of Pharmaceutical Sciences, Soochow University, Kunming 650223, Yunnan, China; 7 Collaborative Innovation Center for Natural Products and Biological Drugs of Yunnan, Kunming 650223, Yunnan, China

## Abstract

There is a constant demand to develop new, effective, and affordable anti-cancer drugs. The traditional Chinese medicine (TCM) is a valuable and alternative resource for identifying novel anti-cancer agents. In this study, we aim to identify the anti-cancer compounds and plants from the TCM database by using cheminformatics. We first predicted 5278 anti-cancer compounds from TCM database. The top 346 compounds were highly potent active in the 60 cell lines test. Similarity analysis revealed that 75% of the 5278 compounds are highly similar to the approved anti-cancer drugs. Based on the predicted anti-cancer compounds, we identified 57 anti-cancer plants by activity enrichment. The identified plants are widely distributed in 46 genera and 28 families, which broadens the scope of the anti-cancer drug screening. Finally, we constructed a network of predicted anti-cancer plants and approved drugs based on the above results. The network highlighted the supportive role of the predicted plant in the development of anti-cancer drug and suggested different molecular anti-cancer mechanisms of the plants. Our study suggests that the predicted compounds and plants from TCM database offer an attractive starting point and a broader scope to mine for potential anti-cancer agents.

Cancer, also known as a malignant tumor, is a group of diseases involving abnormal cell growth with the potential to invade or spread to other parts of the body. The hallmarks of cancer comprise six biological capabilities to support the development of human tumors, which include sustaining proliferative signaling, evading growth suppressors, resisting cell death, enabling replicative immortality, inducing angiogenesis, and activating invasion and metastasis^1^,^2^. Cancer is one of the major causes of death worldwide where the number of cancer patient is in continuous rise. There are over 100 different known cancers that affect humans, and each is classified by the type of cell that is initially affected^3^. In 2012 about 14.1 million new cases of cancer occurred globally (not including skin cancer other than melanoma). It caused about 8.2 million deaths or 14.6% of all human deaths^4^. By 2030, it is predicted that there will be 26 million new cancer cases and 17 million cancer deaths per year^5^.

Today, despite considerable efforts, cancer still remains an aggressive killer worldwide. The most common and highly effective methods of cancer treatment are surgery, chemotherapy and radiotherapy^6^. However, these therapies have numerous limitations and drawbacks^7^. Most cancer patients are diagnosed too late to undergo surgery because of poor diagnosis and other factors. Chemotherapy and radiotherapy have serious side effects and complications such as fatigue, pain, diarrhea, nausea, vomiting, and hair loss^7^. Furthermore, chemotherapy and radiotherapy can result in gradual resistance of cancer cells against treatment^8^.

Therefore there is a constant demand to develop new, effective, and affordable anti-cancer drugs^9^. Medicinal plants constitute a common alternative for cancer treatment in many countries around the world^10^,^11^,^12^,^13^. There are more than 2000 plants used in the traditional Chinese medicine (TCM) according to the TCM database@taiwan (http://tcm.cmu.edu.tw/)^14^. These medicinal plants were used for treatment of various diseases include cancer for thousand years in China^15^,^16^,^17^,^18^,^19^. Many TCM-derived anti-cancer products have been used in western medicine^20^,^21^,^22^,^23^,^24^,^25^,^26^,^27^,^28^. These include vinblastine, vincristine, paclitaxel, camptothecin, epipodophyllotoxin and so on. Vinblastine and vincristine, as the bisindole alkaloids isolated from *Catharanthus roseus*, are the first agents to advance into clinical use for treatment of spleen cancer, liver cancer and childhood leukemia. Paclitaxel, originally isolated from the bark of *Taxus brevifolia*, has also been found in *Taxus chinensis*. It was launched in 1992 and was the best-selling anti-cancer drug in the USA in 2002^8^. Another important class of anti-cancer drugs (topotecan, irinotecan, belotecan, 9-Nitrocamptothecin, and gimatecan) are derived from camptothecin which was isolated from the Chinese ornamental tree *Camptotheca acuminate*^8^,^29^. Epipodophyllotoxin is also an important class of natural product for development of anti-cancer drugs. Etoposide, teniposide and etopophos are semi-synthetic derivatives of epipodophyllotoxin^8^. They are approved for treatment of choriocarcinoma, lung cancer, ovarian and testicular cancers, lymphoma, acute myeloid leukemia, and bladder cancer^6^.

TCM is undoubtedly a valuable resource for identifying novel anti-cancer agents^30^. Regrettably, only a small portion of medicinal plants in the TCM database has been fully phytochemically investigated. It is interest to systematic explore and evaluate the anti-cancer potential of all the plants in the TCM database. However, it is a tedious, expensive and time-consuming process because that it involves screening of large molecular library by experiment. Therefore, the time and money-saving way is that the plants in the TCM database are firstly filtered by the computational analysis of the anti-cancer potential, then evaluated by experiment. The aim of the current investigation is to analyze the anti-cancer potential of all the plants in the TCM database by using cheminformatics, and then identify the anti-cancer compounds and plants from the TCM database in silico. We started with the TCM Database@Taiwan, which is currently the world’s largest non-commercial TCM database^14^. The database contains the relationship between more than 20,000 pure compounds and more than 2000 plants. We first predicted anti-cancer compounds in the database by using our previously published method termed Cancer Drug (CDRUG)^31^. We then determined the anti-cancer plants by performing the anti-cancer activity enrichment analysis (ACEA)^32^. Each of the anti-cancer plants was significantly enriched with anti-cancer compounds. Thus, the identified anti-cancer plants provide important clues and direction for the development of anti-cancer drugs.

## Results

### Prediction of anti-cancer compounds from TCM Database@Taiwan

A total of 21334 compounds from 2402 plants were downloaded from TCM Database@Taiwan. The anti-cancer activity of these compounds was predicted using CDRUG. Finally, a total of 5278 compounds were predicted as anti-cancer compounds (P < 0.05), which is accounting for 25% (5278/21334) of all compounds in the database. Further careful observation, we found the top 346 compounds were identical to those compounds which have been proven active in the 60 cell lines test reported by NCI-60 DTP project^33^. Most of the top 346 compounds have the inhibition rate of growth >50% at less than the dose of 10^−5^ mol/L. The mean logGI50 value (the 50% growth inhibition concentration) of the top 346 compounds is −5.73 with standard deviation 0.89. Among the top 346 compounds, two compounds paclitaxel and homoharringtonine have already been approved for the treatment of various cancers. The logGI50 values of drugs paclitaxel and homoharringtonine are −7.74 and −7.152, respectively.

### Similarity of the predicted anti-cancer compounds with the anti-cancer drugs

Since the compounds identified above were predicted to have anti-cancer activity, we performed a systematic analysis of the similarity between these compounds and the anti-cancer drugs in preclinical, clinical and approved stages from the database of Thomson Reuters Integrity. We got 127, 425 and 219 anti-cancer drugs in preclinical, clinical and approved stages, respectively (**Dataset1** Table S2). Then the similarities of the 5278 compounds against all the anti-cancer drugs of the three types were calculated (see **Methods**). Two compounds are considered structurally similar if their fingerprints have a Tc of 0.70 or greater. We found that 4025 (76%) of the 5278 compounds have similarity (Tc 0.70, MACCS fingerprint) with the anti-cancer drugs in preclinical stage. Similarly, 4406 (83%) and 3952 (75%) of the 5278 compounds have similarity with the anti-cancer drugs in clinical and approved stages, respectively. These results demonstrate the power of CDRUG for prediction of anti-cancer compound. It also shows the importance of these plant-derived compounds in the development of anti-cancer drugs.

### Structural characteristics of the predicted active compounds

**Figure 1:**
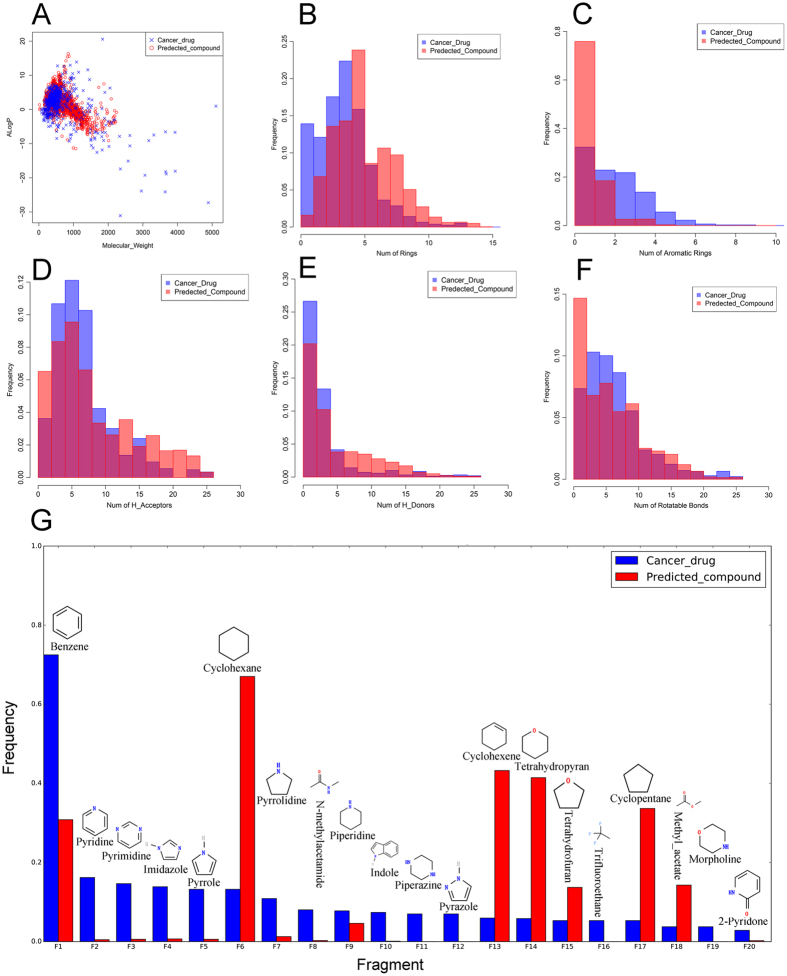
Structural characteristics of the predicted active compounds and cancer drugs. (**A**) Scatter plot of molecular weight against ALogP. (**B**) Histogram plot of the number of rings. (**C**) Histogram plot of the number of aromatic rings. (**D**) Histogram plot of the number of H-bond acceptors. (**E**) Histogram plot of the number of H-bonds donors. (**F**) Histogram plot of the number of rotatable bonds. (**G**) The bar plot of the top 20 common fragments and their frequency (F1-F20). In all plot, the cancer drugs and predicted active compounds were colored by blue and red, respectively.

Orally administered drugs are more likely in areas of chemical space defined by a limited range of molecular properties which were encapsulated in Lipinski’s ‘rule of five’^34^. Lipinski’s rule states that, historically, 90% of orally absorbed drugs had fewer than 5 H-bond donors, less than 10 H-bond acceptors, molecular weight of less than 500 daltons and AlogP values of less than 5. To compare the predicted active compounds with cancer drugs, the four properties and other important properties (number of rotatable bonds, rings, aromatic rings) were calculated in our study (Fig. 1). The distributions of AlogP and molecular weight for the two classes of compounds are highly similar and overlapped (Fig. 1A). In total, 73% of the predicted active compounds have AlogP less than 5 compared with 85% for cancer drugs. In contrast, only 50% and 57% of molecules have a molecular weight less than 500 daltons for the predicted active compounds and cancer drugs, respectively. It suggests the molecules with a molecular weight of more than 500 daltons are also suitable to develop anti-cancer drugs. The major differences between the two classes of compounds emerge when the number of rings and aromatic rings is considered (Fig. 1B,C). 40% of the predicted active compounds have five or more rings compared with 18% for the cancer drugs. Conversely, only 6% of the predicted active compounds have two or more aromatic rings compared with 40% for the cancer drugs. The ratios of the number of rings and aromatic rings are 8.39:1 and 1.67:1 for the predicted active compounds and cancer drugs, respectively. The predicted active compounds tend toward a high ratio of the number of rings and aromatic rings compared with the cancer drugs. The distributions of the other three molecular properties (number of H-bond donors, H-bond acceptors and rotatable bonds) are similar between the two classes of compounds (Fig. 1D–F).

To further compare the two classes of compounds, the most common fragments and their frequency for these molecules were analyzed. The top 20 common fragments in the cancer drugs were shown in the Fig. 1G. The frequency of these fragments is very different between the two classes of compounds. The frequency of most fragments in the predicted active compounds is less than that in the cancer drugs. For example, the frequency of pyridine, pyrimidine, imidazole, pyrrole and pyrrolidine in the predicted active compounds is extremely low. It is noteworthy that the fragments piperazine, pyrazole, trifluoroethane and morpholine are even absent in the predicted active compounds. Only six fragments cyclohexane, cyclohexene, tetrahydropyran, tetrahydrofuran, cyclopentane and methyl acetate have higher frequency in the predicted active compounds. The analysis of molecular properties above suggested the predicted active compounds tended toward a high ratio of rings and aromatic rings. This tendency also emerges in the fragments analysis. 73% of the cancer drugs have unsaturated rings benzene. In contrast, 67% of the predicted active compounds have saturated ring cyclohexane. The number of unsaturated rings in the predicted active compounds is far less than that in the cancer drugs. And the number of saturated rings in the predicted active compounds is far more than that in the cancer drugs.

### Identification of anti-cancer plants

We have predicted thousands of compounds with anti-cancer activity above. It is worth to identify the plant which is enriched with anti-cancer compounds. The identification of anti-cancer plants is of great value in the introduction, utilization and protection of medicinal plants. It is also important in the development of anti-cancer drugs. Therefore, based on the predicted anti-cancer compounds, we identified 57 anti-cancer plants (P_adj < 0.05) (Table 1) using the method named ACEA. These plants belong to 46 genera and 28 families. Detailed information concerning the anti-cancer plants can be found in Supplementary Dataset1 Table S3. When checked the family distribution of these plants, we have noticed that the anti-cancer plants were more frequent from the families Araliaceae, Asteraceae, Boraginaceae, Ranunculaceae and Rosaceae. For example, there are 8 anti-cancer plants belonged to family Araliaceae. They are *Panax bipinnatifidum Seem*., *Panax japonicus*, *Panax notoginseng*, *Panax quinquefolium L*., *Panax ginseng*, *Aralia elata*, *Oplopanax elatus Nakai*, *Aralia taibaiensis*. These plants have potential ability to kill cancer cells due to the enrichment of anti-cancer compounds. To verify this result, we performed literature survey using Thomson Reuters Web of Science database. We found that many of these plants have been reported to have anti-cancer activity in several studies, such as *Salvia miltiorrhiza*, *Paris polyphylla, Gynostemma pentaphyllum*, *Panax ginseng*, *Panax notoginseng*, *Brucea javanica*, *Platycodon grandiflorum*. Of these plants, *Salvia miltiorrhiza* is the most studied plant for cancer treatment. There are 84 predicted anti-cancer compounds derived from *Salvia miltiorrhiza*. These compounds showed potent activities against various types of cancer including esophageal cancer, gastric cancer, colon cancer, liver cancer, prostate cancer and breast cancer^35^,^36^,^37^,^38^,^39^. Another more studied plant is *Paris polyphylla Smith* which contains 13 predicted anti-cancer compounds. *Paris polyphylla Smith* has been studied for the treatment of breast cancer, gastric cancer and lung cancer^40^,^41^,^42^,^43^. Notably, there are 24 identified anti-cancer plants which were little studied before. These new identified anti-cancer plants are worthy of further studies and provide more chances for the development of cancer drug.

### Network of predicted anti-cancer plants and anti-cancer drugs

**Figure 2:**
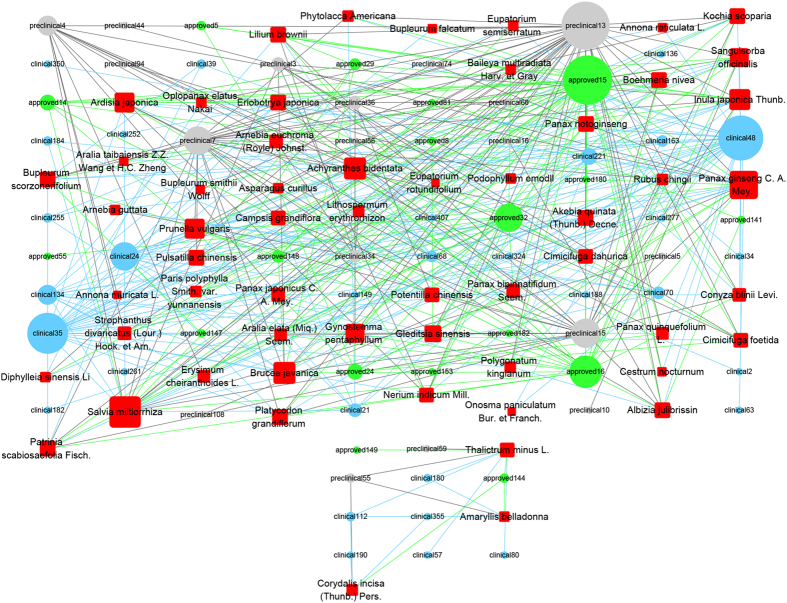
Network of predicted anti-cancer plants and anti-cancer drugs . The network connects plant and drug if the compounds in this plant show similarity with this drug (Tc 0.70, MACCS fingerprint). Two isolated sub-networks were shown in the figure. The red rectangle, green circle, light blue circle and gray circle represent predicted anti-cancer plant, approved drug, clinical drug and preclinical drug, respectively. The lines link the approved drug, clinical drug and preclinical drug are color as green, light blue and gray, respectively. The node size is proportional to the number of connections.

To show how extend the predicted anti-cancer plants to support the development of anti-cancer drugs, we constructed a network of predicted anti-cancer plants and anti-cancer drugs based on the results above using Cytoscape v3.2. The network connects plant and drug if the compounds in this plant show similarity with this drug (Tc 0.70, MACCS fingerprint). It generated a network which contains 57 plants and 67 anti-cancer drugs (Fig. 2). This network highlights the supportive role of these plants in the development of cancer drugs. All the predicted anti-cancer plants associate with the development of cancer drugs. Some of them appear to be more important and closely related to the development of anti-cancer drugs, such as *Salvia miltiorrhiza*, *Panax ginseng C. A. Mey*, *Brucea javanica*, and *Achyranthes bidentata*. *Salvia miltiorrhiza* connected 6 approved drugs, 10 clinical drugs and 8 preclinical drugs. The six approved drugs are 4-Hydroxyandrostenedione, prednisolone, 17-Methyltestosterone, megestrol acetate, methylprednisolone sodium succinate and bexarotene. These drugs have been used for treatment of breast cancer, lymphoma. Bexarotene is being developed in clinical phase II for treating non-small cell lung cancer. *Panax ginseng C. A. Mey* connected 6 approved drugs, 9 clinical drugs and 6 preclinical drugs. One of the clinical drugs, clinical35 is identical to Ginsenoside K (TC = 1) which exist in *Panax ginseng C. A. Mey*. Ginsenoside K is a steroidal saponin in phase I clinical studies at IL-HWA for the treatment of cancer. Similarly, *Brucea javanica* connected 5 approved drugs, 4 clinical drugs and 6 preclinical drugs. *Achyranthes bidentata* connected 4 approved drugs, 6 clinical drugs and 5 preclinical drugs.

Surprisingly, two isolated sub-networks were found in the overall network. The two sub-networks are involved in different drugs, thus maybe different molecular mechanism of anti-cancer. The smaller sub-network contains three plants (*Corydalis incisa*, *Amaryllis belladonna*, and *Thalictrum minus L*) and two approved drugs (approved144: homoharringtonine and approved149: bosutinib). Homoharringtonine was originally isolated from Chinese tree *Cephalotaxus harringtonia*^44^. The three plants and *Cephalotaxus harringtonia* are distributed in different family and order. The diversity of plants and compounds suggests the three plants may provide an alternative resource for discovery of new compounds with activity similar to homoharringtonine. Further studies should be performed to screen the three plants.

## Discussion

With the aim of systematic explore and evaluate the anti-cancer potential of all the plants in the TCM database, we identified 5278 anti-cancer compounds in this study. The predicted anti-cancer compounds account for 25% (5278/21334) of all compounds in the database. After calculating similarity, 3952 (75%) of the 5278 compounds have similarity with the approved anti-cancer drugs (Tc 0.70, MACCS fingerprint). It suggests the great value of these predicted anti-cancer compounds. Some new similar drugs may be discovered from these compounds. As natural products, these compounds show less side effects compared with synthetic compound. These compounds can be a ready and effective anti-cancer molecular library. Further experiments should design to screen the library to found the drugs with more active but less side effects.

The compounds which have similarity with the approved anti-cancer drugs can be used to develop me-too drugs. And its opposite, the innovative drugs are developed by using structurally dissimilar compounds and different molecular mechanism. There are about 25% of the 5278 compounds have no similarity with all the anti-cancer drugs in preclinical, clinical and approved stages from the database of Thomson Reuters Integrity. With the frequent use of anti-cancer drugs and increased duration of treatment, cancer cell may be resistant to the drugs. The problem of drug resistance can be shoveled by developing new and effective anti-cancer drugs. Therefore, these structurally dissimilar compounds are promising molecules and can be used to develop innovative drugs.

Lipinski’s rule is often used to determine if a chemical compound with a certain pharmacological activity has properties that would make it a likely orally active drug in humans. The rule evaluates drug-likeness by using four molecular properties (ALogP, molecular weight, H-bond acceptors, and H-bonds donors). The analysis of molecular properties revealed that the distributions of ALogP, molecular weight, H-bond acceptors, and H-bonds donors are very similar and overlapped between the predicted active compounds and cancer drugs. The distribution of rotatable bonds is also similar between the two classes of compounds. These results suggested that most of the predicted active compounds have a good drug-likeness. However, we found that the frequency of most common fragments is very different between the two classes of compounds. Both fragment analysis and molecular property analysis revealed that the ratio of rings and aromatic rings tended to become smaller from the predicted active compounds to cancer drugs. Saturated rings are enriched in the predicted active compounds and unsaturated rings are enriched in the cancer drugs. Generally, unsaturated compounds are more reactive than saturated compounds^45^. Therefore, the reactivity of the predicted active compounds may be lower compared with the cancer drugs. As the degree of reactivity links the level of toxic side effect^46^, our results suggested the lower toxicity of the predicted active compounds. In addition, trifluoroethane fragment, a toxic substance, is common in the cancer drugs but absent in the predicted active compounds. It also suggested the lower toxicity of the predicted active compounds.

In our study, we identified 57 anti-cancer plants using the ACEA method which based on the enrichment of anti-cancer compounds in corresponding plant. Literature survey showed that many of these plants have been reported to have anti-cancer activity in several studies, such as Salvia miltiorrhiza, Paris polyphylla, Gynostemma pentaphyllum, Panax ginseng, Panax notoginseng, Brucea javanica, Platycodon grandiflorum. Notably, there are 24 identified anti-cancer plants which were little studied before. Of these plants, 14 plants belong to the families in which many species have already been reported as anti-cancer plants. In contrast, the other 10 plants belong to the families in which only a few species have been studied as anti-cancer plants, such as caprifoliaceae, solanaceae, bignoniaceae, brassicaceae. The identified plants are widely distributed in 46 genera and 28 families. The identification of these genera and families provides a broader scope and vision for the screening of anti-cancer drugs. These new identified anti-cancer plants are worthy of further studies and provide more chances for the development of cancer drug. Our results may contribute to decision-making in the process of introduction, protection and utilization of medicinal plants. This information of the anti-cancer plants can improve the rationality of decision-making about introduction of medicinal plants.

The prediction of anti-cancer plants requires the annotation information of plant and the compounds in corresponding plant. Incomplete information may affect the results of prediction. For example, there are close to half of 2402 plans which have less than 5 compounds annotated in corresponding plant. Therefore, these plants can not be identified using the ACEA method. Our study mainly based on the TCM Database@Taiwan, which is currently the world’s largest and most comprehensive TCM database. With the increasing information in database, the predicted results will be more accurate.

After generation of the plants-drugs network, we found two isolated sub-networks in the overall network. The two sub-networks may be involved in different molecular mechanism of anti-cancer due to connecting different drugs. The smaller sub-network contains two approved drugs (approved144: homoharringtonine and approved149: bosutinib). The bigger sub-network contains 16 approved drugs. In order to probe the molecular mechanisms, we got the target information of these drugs from DrugBank. We found the drugs in the smaller network can bind to the ribosome and inhibit polypeptide chain elongation, thus inhibit protein synthesis. In contrast, the drugs in the bigger network are mainly involved in two molecular mechanism. One is regulation of nuclear receptors and estrogen-related signal. The other is inhibition of DNA replication. Therefore, this result suggests that medicinal plants may exert anti-cancer activity by different molecular mechanism. The plants-drugs network can be used for exploration of molecular mechanism of anti-cancer.

With the accumulation of biological data and increase of the variety and complexity of data types, bioinformatics and cheminformatics play an important role in the integration of these data. Until now, there are two types of data are useful and available for data-mining biologically active compound. One is experimental biological activity data including high-throughput chemical biology screening datasets in Pubchem database^47^, such as anti-cancer biological activity data, anti-HIV biological activity data and anti-tuberculosis biological activity data. The other is the curated data about TCM plants and their derived ingredients in several TCM database. The two types of data offer a new opportunity to mine for potential compounds with various activities by using bioinformatics and cheminformatics^48^,^49^,^50^. Salma *et al*. identified anti-tubercular compounds from TCM by integrating anti-tuberculosis biological activity data and TCM related data^50^. Kenneth *et al*. identified quinone subtypes effective against melanoma and leukemia cell by data-mining the GI50 values of the NCI cancer cell line compound^51^. Thomas *et al*. used random forest to virtual screen Chinese herbs for potential inhibitors against several therapeutically important molecular targets^52^.

In summary, our analysis suggests that the predicted compounds and plants from TCM database offer an attractive starting point and a broader scope to mine for potential anti-cancer agents. We hope that this study would accelerate in-depth analysis and discovery of anti-cancer agents from TCM.

## Methods

To infer anti-cancer plants, we first collected the information concerning the plants and the plant-derived compounds from the TCM Database@Taiwan. The relationship of the pant and its derived compounds was also collected. All compounds were downloaded as mol2 (3D) format. The format was converted to SMILES string^53^ by the Open Babel toolbox^54^. A total of 2402 plants and 21334 compounds were collected and downloaded for further study. Detailed information concerning the plants and all compounds can be found in Supplementary Dataset1 Table S1.

The anti-cancer activities of all the compounds were predicted using CDRUG, which was developed by our laboratory^31^. CDRUG uses a novel molecular description method (relative frequency-weighted fingerprint) and a hybrid score to measure the similarity between the query and the active compounds. Then a confidence level (P-value) is calculated to predict whether a compound has anti-cancer activity. The performance analysis shows that CDRUG has the area under curve of 0.878 and can hit 65% positive results at the false-positive rate of 0.05. Thus CDRUG is effective to predict anti-cancer activity of the chemical compounds. In this study, we used the default (P < 0.05) cutoff in CDRUG to screen the 21334 compounds in the TCM Database@Taiwan.

After anti-cancer activity prediction of the 21334 compounds, we measured whether a plant has potential ability to kill cancer cells using the method named ACEA^32^. ACEA is based on the results of anti-cancer activity prediction and uses a hypergeometric distribution to perform enrichment analysis. The P-value of each plant can be calculated using the following equation:


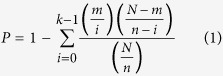


Here, N and n are the total number of compounds and the total number of anti-cancer compounds in the TCM Database@Taiwan, respectively; m and k represent the number of compounds and the number of anti-cancer compounds in a plant, respectively. Both n and k are calculated using CDRUG. Because multiple tests (2402 plants) were performed, the Bonferroni correction method was used to adjust the P-value determined by ACEA:


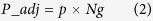


Here, P_adj is the adjusted P-value of ACEA, P is the P-value of ACEA (without Bonferroni correction) and Ng is the number of plants in the TCM Database@Taiwan. Only plants with P_adj < 0.05 were retained.

In order to compare the similarity of the predicted anti-cancer compounds with the anti-cancer drugs in the different development stages, we got the information concerning the anti-cancer drugs in preclinical, clinical and approved stages from the database of Thomson Reuters Integrity (www.thomsonreutersintegrity.com). The molecular properties of the predicted active compounds and anti-cancer drugs were calculated using the protocol ‘Calculate Molecular Properties’ in Pipeline Pilot v8.5^55^. The calculated properties include ALogP, molecular weight, and the number of rotatable bonds, rings, aromatic rings, H-bond acceptors, and H-bonds donors, and so on. Detailed information and molecular properties for the predicted active compounds and anti-cancer drugs can be found in Supplementary Dataset1 Table S2. The most common fragments and their frequency were calculated using the protocol ‘Most Frequent Fragments’ Pipeline Pilot v8.5. These fragments and their frequency are available in Supplementary Dataset1 Table S4. The structural similarity was measured by Tanimoto coefficient (Tc)^56^. Tc is defined as Tc = C(i, j)/U(i, j), where C(i, j) is the number of common features in the fingerprints of molecules i and j and where U(i, j) is the number of all features in the union of the fingerprints of molecules i and j. The fingerprint MACCS implemented in the Pybel^57^ were generated for each structure and used to calculate TC. Two compounds are considered structurally similar if their fingerprints have a Tc of 0.70 or greater^58^,^59^. After calculation, the similarity network was visualized using Cytoscape v3.2^60^.

## Additional Information

**How to cite this article**: Dai, S.-X. *et al*. In silico identification of anti-cancer compounds and plants from traditional Chinese medicine database. *Sci. Rep*. **6**, 25462; doi: 10.1038/srep25462 (2016).

## Supplementary Material

Supplementary Information

## Figures and Tables

**Table 1:** The predicted anti-cancer plants.

Plant_name	Family	compound	P_adj	literature
Gynostemma pentaphyllum	Cucurbitaceae	36	3.91E-48	39
Platycodon grandiflorum	Campanulaceae	11	2.69E-28	15
Panax japonicus C. A. Mey.	Araliaceae	8	3.65E-26	3
Panax bipinnatifidum Seem.	Araliaceae	42	3.65E-26	0
Panax notoginseng	Araliaceae	14	4.33E-25	28
Annona muricata L.	Annonaceae	39	4.41E-19	10
Pulsatilla chinensis	Ranunculaceae	12	1.70E-14	2
Salvia miltiorrhiza	Lamiaceae	8	6.12E-13	63
Panax quinquefolium L.	Araliaceae	13	1.72E-12	4
Prunella vulgaris	Lamiaceae	13	5.62E-12	13
Polygonatum kingianum	Asparagaceae	50	2.26E-11	0
Patrinia scabiosaefolia	Caprifoliaceae	50	4.69E-11	0
Campsis grandiflora	Bignoniaceae	94	1.05E-10	0
Albizia julibrissin	Fabaceae	32	2.75E-10	0
Gleditsia sinensis	Fabaceae	76	6.61E-10	9
Bupleurum scorzonerifolium	Apiaceae	15	1.07E-08	3
Ardisia japonica	Primulaceae	12	1.68E-08	2
Achyranthes bidentata	Amaranthaceae	8	4.17E-08	4
Sanguisorba officinalis	Rosaceae	27	4.99E-07	14
Cimicifuga foetida	Ranunculaceae	36	7.99E-07	7
Arnebia guttata	Boraginaceae	14	7.99E-07	0
Diphylleia sinensis Li	Berberidaceae	16	1.88E-06	0
Erysimum cheiranthoides L.	Brassicaceae	11	1.88E-06	0
Cimicifuga dahurica	Ranunculaceae	14	6.07E-06	0
Asparagus curillus	Asparagaceae	9	7.63E-06	0
Rubus chingii	Rosaceae	8	7.63E-06	0
Podophyllum emodll	Berberidaceae	15	1.15E-05	0
Lithospermum erythrorhizon	Boraginaceae	18	1.70E-05	2
Strophanthus divaricatus	Apocynaceae	22	2.32E-05	0
Panax ginseng C. A. Mey.	Araliaceae	15	3.09E-05	31
Aralia elata (Miq.) Seem.	Araliaceae	15	1.46E-04	8
Potentilla chinensis	Rosaceae	17	1.51E-04	1
Nerium indicum Mill.	Apocynaceae	9	2.04E-04	1
Paris polyphylla Smith	Melanthiaceae	16	3.33E-04	47
Annona reticulata L.	Annonaceae	66	3.33E-04	1
Phytolacca Americana	Phytolaccaceae	28	5.42E-04	2
Boehmeria nivea	Urticaceae	36	7.12E-04	1
Conyza blinii Levi.	Asteraceae	31	7.75E-04	0
Cestrum nocturnum	Solanaceae	104	2.53E-03	0
Brucea javanica	Simaroubaceae	84	2.55E-03	22
Kochia scoparia	Amaranthaceae	53	3.34E-03	2
Baileya multiradiata	Asteraceae	14	4.70E-03	0
Arnebia euchroma	Boraginaceae	9	4.70E-03	2
Thalictrum minus L.	Ranunculaceae	13	6.24E-03	0
Inula japonica Thunb.	Asteraceae	18	8.21E-03	2
Akebia quinata	Lardizabalaceae	19	8.21E-03	10
Onosma paniculatum	Boraginaceae	41	8.21E-03	2
Lilium brownii	Liliaceae	37	8.30E-03	0
Eupatorium semiserratum	Asteraceae	25	8.30E-03	0
Corydalis incisa	Papaveraceae	14	8.30E-03	0
Eriobotrya japonica	Rosaceae	14	1.22E-02	3
Oplopanax elatus Nakai	Araliaceae	36	1.55E-02	1
Bupleurum falcatum	Apiaceae	17	2.62E-02	1
Aralia taibaiensis	Araliaceae	16	2.62E-02	0
Amaryllis belladonna	Amaryllidaceae	40	3.36E-02	0
Eupatorium rotundifolium	Asteraceae	66	3.36E-02	0
Bupleurum smithii Wolff	Apiaceae	15	3.36E-02	0

The third column represents the number of compounds with anticancer activity in this plant. The last column represents the number of literature and patent whose titles contain both words “the name of plant” and “cancer”.
