# Corrigendum: In silico identification of anti-cancer compounds and plants from traditional Chinese medicine database

**DOI:** 10.1038/srep34972

**Published:** 2016-10-10

**Authors:** Shao-Xing Dai, Wen-Xing Li, Fei-Fei Han, Yi-Cheng Guo, Jun-Juan Zheng, Jia-Qian Liu, Qian Wang, Yue-Dong Gao, Gong-Hua Li, Jing-Fei Huang

Scientific Reports 6: Article number: 25462; 10.1038/srep25462 published online: 05 05 2016; updated: 10 10 2016.

In this Article, the values given in the column labeled ‘compound’ of Table 1 are incorrect. The correct Table 1 appears below.

## Figures and Tables

**Table 1:** 

Plant_name	Family	compound	P_adj	literature
Gynostemma pentaphyllum	Cucurbitaceae	104	3.91E-48	39
Platycodon grandiflorum	Campanulaceae	66	2.69E-28	15
Panax japonicus C. A. Mey.	Araliaceae	50	3.65E-26	3
Panax bipinnatifidum Seem.	Araliaceae	50	3.65E-26	0
Panax notoginseng	Araliaceae	94	4.33E-25	28
Annona muricata L.	Annonaceae	42	4.41E-19	10
Pulsatilla chinensis	Ranunculaceae	37	1.70E-14	2
Salvia miltiorrhiza	Lamiaceae	84	6.12E-13	63
Panax quinquefolium L.	Araliaceae	32	1.72E-12	4
Prunella vulgaris	Lamiaceae	53	5.62E-12	13
Polygonatum kingianum	Asparagaceae	27	2.26E-11	0
Patrinia scabiosaefolia	Caprifoliaceae	28	4.69E-11	0
Campsis grandiflora	Bignoniaceae	22	1.05E-10	0
Albizia julibrissin	Fabaceae	36	2.75E-10	0
Gleditsia sinensis	Fabaceae	31	6.61E-10	9
Bupleurum scorzonerifolium	Apiaceae	39	1.07E-08	3
Ardisia japonica	Primulaceae	41	1.68E-08	2
Achyranthes bidentata	Amaranthaceae	36	4.17E-08	4
Sanguisorba officinalis	Rosaceae	36	4.99E-07	14
Cimicifuga foetida	Ranunculaceae	25	7.99E-07	7
Arnebia guttata	Boraginaceae	15	7.99E-07	0
Diphylleia sinensis Li	Berberidaceae	15	1.88E-06	0
Erysimum cheiranthoides L.	Brassicaceae	16	1.88E-06	0
Cimicifuga dahurica	Ranunculaceae	14	6.07E-06	0
Asparagus curillus	Asparagaceae	14	7.63E-06	0
Rubus chingii	Rosaceae	17	7.63E-06	0
Podophyllum emodll	Berberidaceae	18	1.15E-05	0
Lithospermum erythrorhizon	Boraginaceae	15	1.70E-05	2
Strophanthus divaricatus	Apocynaceae	13	2.32E-05	0
Panax ginseng C. A. Mey.	Araliaceae	76	3.09E-05	31
Aralia elata (Miq.) Seem.	Araliaceae	15	1.46E-04	8
Potentilla chinensis	Rosaceae	16	1.51E-04	1
Nerium indicum Mill.	Apocynaceae	13	2.04E-04	1
Paris polyphylla Smith	Melanthiaceae	13	3.33E-04	47
Annona reticulata L.	Annonaceae	14	3.33E-04	1
Phytolacca Americana	Phytolaccaceae	19	5.42E-04	2
Boehmeria nivea	Urticaceae	18	7.12E-04	1
Conyza blinii Levi.	Asteraceae	16	7.75E-04	0
Cestrum nocturnum	Solanaceae	15	2.53E-03	0
Brucea javanica	Simaroubaceae	66	2.55E-03	22
Kochia scoparia	Amaranthaceae	11	3.34E-03	2
Baileya multiradiata	Asteraceae	11	4.70E-03	0
Arnebia euchroma	Boraginaceae	17	4.70E-03	2
Thalictrum minus L.	Ranunculaceae	14	6.24E-03	0
Inula japonica Thunb.	Asteraceae	14	8.21E-03	2
Akebia quinata	Lardizabalaceae	14	8.21E-03	10
Onosma paniculatum	Boraginaceae	9	8.21E-03	2
Lilium brownii	Liliaceae	9	8.30E-03	0
Eupatorium semiserratum	Asteraceae	9	8.30E-03	0
Corydalis incisa	Papaveraceae	18	8.30E-03	0
Eriobotrya japonica	Rosaceae	40	1.22E-02	3
Oplopanax elatus Nakai	Araliaceae	12	1.55E-02	1
Bupleurum falcatum	Apiaceae	12	2.62E-02	1
Aralia taibaiensis	Araliaceae	8	2.62E-02	0
Amaryllis belladonna	Amaryllidaceae	8	3.36E-02	0
Eupatorium rotundifolium	Asteraceae	8	3.36E-02	0
Bupleurum smithii Wolff	Apiaceae	8	3.36E-02	0

